# Assessing Therapeutic Efficacy in Real-time by Hyperpolarized Magnetic Resonance Metabolic Imaging

**DOI:** 10.3390/cells8040340

**Published:** 2019-04-11

**Authors:** Prasanta Dutta, Travis C. Salzillo, Shivanand Pudakalakatti, Seth T. Gammon, Benny A. Kaipparettu, Florencia McAllister, Shawn Wagner, Daniel E. Frigo, Christopher J. Logothetis, Niki M. Zacharias, Pratip K. Bhattacharya

**Affiliations:** 1 Department of Cancer Systems Imaging, The University of Texas MD Anderson Cancer Center, Houston, TX 77030, USA; pdutta@mdanderson.org (P.D.); TCSalzillo@mdanderson.org (T.C.S.); SPudakalakatti@mdanderson.org (S.P.); STGammon@mdanderson.org (S.T.G.); Frigo@mdanderson.org (D.E.F.); NMZacharias@mdanderson.org (N.M.Z.); 2 The University of Texas MD Anderson Cancer Center UT Health Graduate School of Biomedical Sciences, Houston, TX 77030, USA; 3 Department of Molecular and Human Genetics, Baylor College of Medicine, Houston, TX 77030, USA; kaippare@bcm.edu; 4 Department of Clinical Cancer Prevention, The University of Texas MD Anderson Cancer Center, Houston, TX 77030, USA; FMcAllister@mdanderson.org; 5 Biomedical Imaging Research Institute Cedars Sinai Medical Center, Los Angeles, CA 90048, USA; shawn.wagner@cshs.org; 6 Department of Genitourinary Medical Oncology, The University of Texas MD Anderson Cancer Center, Houston, TX 77030, USA; clogothe@mdanderson.org; 7 Department of Clinical Therapeutics, University of Athens, 11527 Athens, Greece; 8 Department of Urology, The University of Texas MD Anderson Cancer Center, Houston, TX 77030, USA

**Keywords:** cancer metabolism, hyperpolarization, MRI, therapy monitoring, metabolic imaging

## Abstract

Precisely measuring tumor-associated alterations in metabolism clinically will enable the efficient assessment of therapeutic responses. Advances in imaging technologies can exploit the differences in cancer-associated cell metabolism as compared to normal tissue metabolism, linking changes in target metabolism to therapeutic efficacy. Metabolic imaging by Positron Emission Tomography (PET) employing 2-fluoro-deoxy-glucose ([^18^F]FDG) has been used as a routine diagnostic tool in the clinic. Recently developed hyperpolarized Magnetic Resonance (HP-MR), which radically increases the sensitivity of conventional MRI, has created a renewed interest in functional and metabolic imaging. The successful translation of this technique to the clinic was achieved recently with measurements of ^13^C-pyruvate metabolism. Here, we review the potential clinical roles for metabolic imaging with hyperpolarized MRI as applied in assessing therapeutic intervention in different cancer systems.

## 1. Introduction

**Figure 1:**
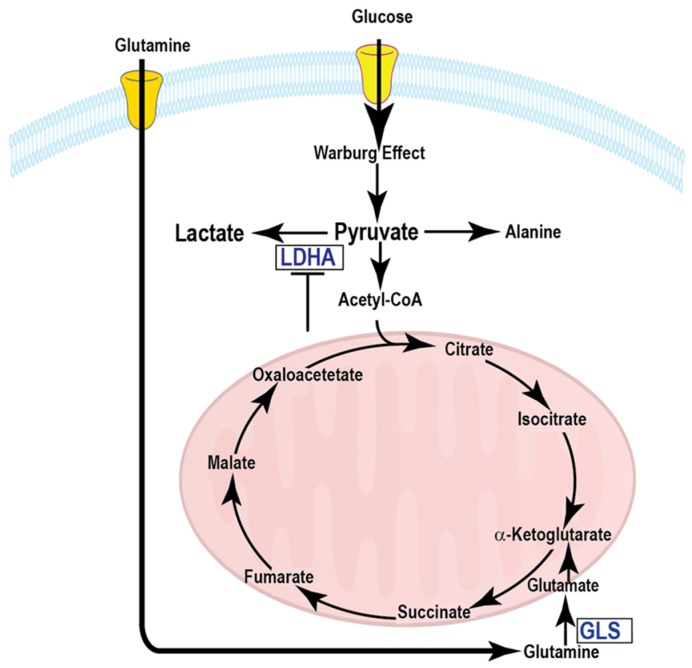
A schematic of glycolysis and Krebs cycle metabolic pathways.

Aberrant metabolism is one of the hallmarks of cancer [1]. Cancer cells reprogram metabolism to sustain rapid proliferation and survival. The alteration of glucose metabolism in cancer is known as aerobic glycolysis or the Warburg Effect [2], which describes the ability of cancer cells to avidly metabolize glucose to lactate, even in the presence of oxygen. A schematic of glycolysis and Krebs cycle metabolic pathways is shown in Figure 1. A manifestation of the Warburg Effect is the increased 2-[^18^F]fluoro-2-deoxy-d-glucose ([^18^F]FDG) uptake by many solid tumors as determined by positron emission tomographic (PET) scans. [^18^F]FDG-PET is the most widely used metabolic imaging technique in the clinic for tumor staging and assessment of treatment response to date [3]. This is the first approved imaging technology that integrates biologic and anatomic factors in clinical assessments. However, the [^18^F]FDG-PET imaging modality suffers from a lack of specificity, as increased glucose uptake also occurs in inflammation and normal brain tissue. Another drawback of PET is that only the radiotracer uptake can be assessed and downstream metabolites cannot be tracked dynamically.

There is a pressing need to develop noninvasive, nonradioactive imaging techniques for cancer diagnosis and monitoring tumor treatment response in the clinic for the appropriate selection of effective therapies. Hyperpolarized magnetic resonance (HP-MR) provides a >10,000-fold signal enhancement relative to conventional MR. HP-MR is capable of detecting endogenous metabolic substrates in real-time to monitor in vivo metabolic fluxes through the multiple key biochemical pathways including glycolysis and Krebs cycle [4]. In particular, conversion of hyperpolarized ^13^C-labeled pyruvate to lactate, catalyzed by lactate dehydrogenase (LDH), has been shown to have a number of potential applications, such as diagnosis, staging tumor grade and monitoring therapy response [5,6,7,8,9]. [2-^13^C]-fructose has been hyperpolarized and tested in in vivo metabolic imaging that demonstrated a difference in uptake and metabolism in regions of tumor relative to surrounding tissue [10]. In this review, we briefly discuss the application of the state-of-the-art hyperpolarization technique to assess treatment response, current challenges, and future directions to make HP-MR a routine and viable diagnostic imaging modality in the clinic.

## 2. State-of-the-Art Hyperpolarization Techniques

**Figure 2:**
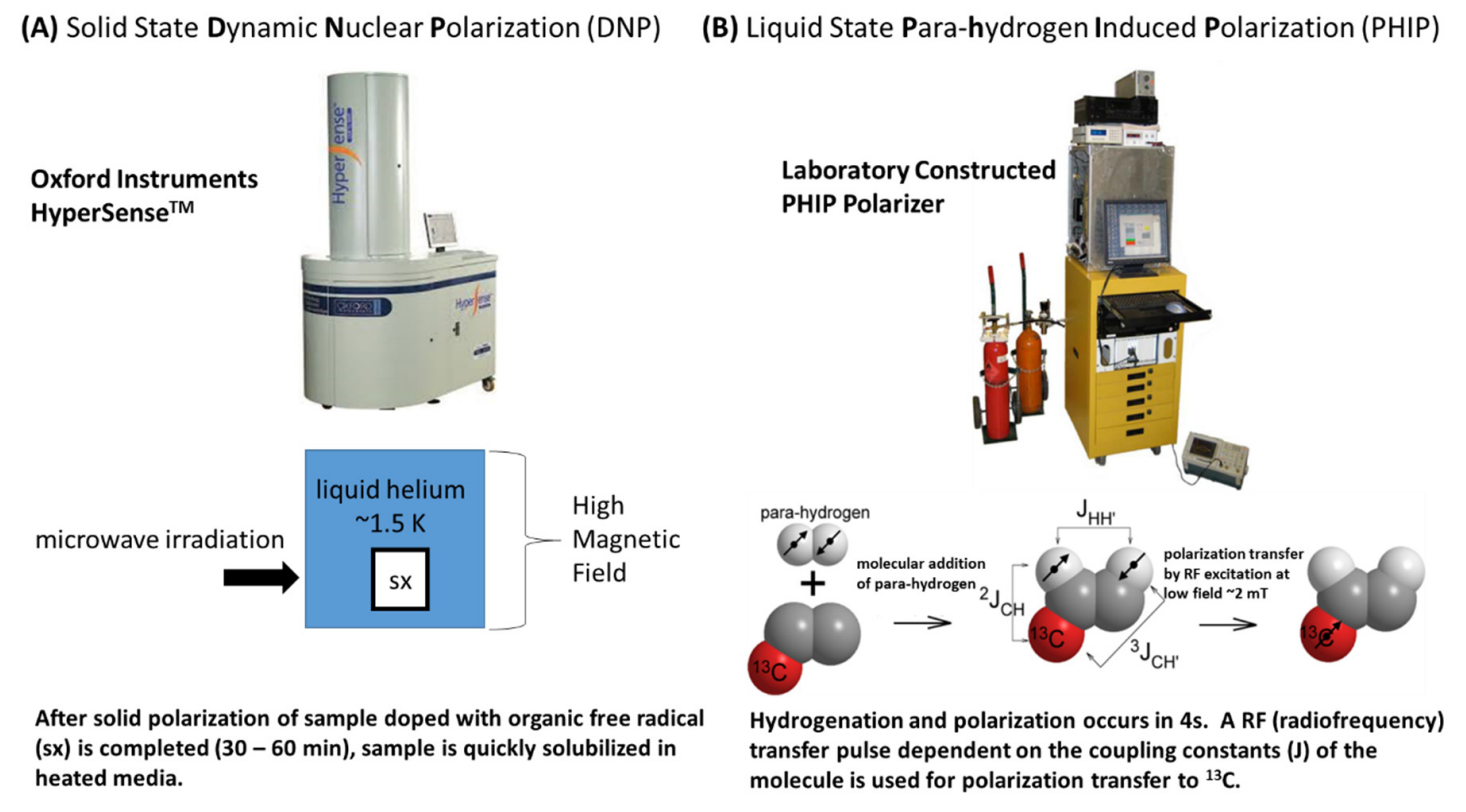
Schematic illustration of (**A)** DNP and (**B**) PHIP techniques.

Conventional MR always suffers from its poor signal sensitivity. Dynamic nuclear polarization (DNP) is a solid-state technique that addresses this problem by significantly increasing the signal-to-noise ratio. Briefly, ^13^C or ^15^N labeled molecules, doped with small quantities of a stable radical, are cooled to approximately 1 K in a high magnetic field (3–5T); microwave irradiation transfers polarization from the fully polarized electron spins on the radical to the ^13^C or ^15^N nuclei (Figure 2A). Once the sample reaches the hyperpolarized state, it is then rapidly dissolved using a hot pressurized solution, which can be injected into an animal or human subjects in an imaging magnet. The increase in signal-to-noise ratio with DNP-MR is between 10^4^ and 10^5^, allowing the detection of not only the substrate and products but also of their spatial distribution using spectroscopic imaging [11]. The technique has been used to image ^13^C containing metabolites in tumors, cardiac tissue, and brain. The key advantage of DNP-MR is that both the injected substrate and its metabolic products can be detected, allowing real-time observation of multiple metabolites. In addition, multiple hyperpolarized molecules can be detected simultaneously, allowing several metabolic pathways to be probed as generated in real time [12]. In addition to DNP, Parahydrogen Induced Polarization (PHIP) is a liquid state hyperpolarization technique that has the potential for in vivo metabolic imaging applications [13]. In the PHIP method, nuclear polarization is increased through a chemical reaction involving a parahydrogen state where the hydrogen nuclei are oriented such that their net magnetization cancels each other (Figure 2B). The non-equilibrium spin order of the parahydrogen molecule is converted to nuclear polarization of the ^13^C nucleus in the substrate by hydrogenation [14]. The range of compounds hyperpolarized by PHIP with metabolic imaging potential is limited; nonetheless, the cost associated with PHIP is substantially lower than DNP and therefore this polarization technique could have great promise in specific applications [15,16,17]. Recently, the SABRE (Signal Amplification by Reversible Exchange) technique have also shown promise for in vivo imaging applications [18].

## 3. Assessment of Different Therapeutic Interventions

Clinical assessments of tumor responses to treatment are still based largely on observed changes in tumor size or uptake of [^18^F]FDG. However, this might not always be appropriate, particularly for the detection of early response or if the drug does not result in tumor shrinkage, as in the case of antiangiogenic drugs. Additionally, treatment assessment using metabolic [^18^F]FDG-PET imaging is difficult in some organs, e.g., prostate and brain, due to both low tumor uptake and increased background uptake, respectively. Evaluation of treatment response is likely to be the clinical scenario where hyperpolarized [1-^13^C]pyruvate will have the greatest impact, as it could lead to immediate changes in clinical management, allowing the clinician to change a non-responding patient to a more effective drug at an early stage [19]. Early assessment of treatment response could also be used to accelerate the introduction of new drugs into the clinic indicating drug efficacy in early-stage clinical trials. In support of this concept, numerous studies have shown early decreases in the hyperpolarized ^13^C-labeled exchange between injected [1-^13^C]pyruvate and the endogenous lactate pool in a range of cancer models following treatment with chemotherapy, targeted therapy, radiotherapy and recently, with immunotherapy. Each of these applications is systematically described in the following paragraphs. The early treatment response has been detected by measuring the rate of LDH-catalyzed lactate flux change in tumors after intravenously delivering HP-pyruvate [19]. It has been claimed that the transfer of this technique to the clinic may allow an oncologist to determine whether a cancer is responding to the treatment within hours of delivery. If the tumor is not responding, a more effective treatment regimen could then be initiated. This timely decision-making approach will have a significant impact on the drug development process and may enable a more personalized approach to therapy.

### 3.1. Metabolically Targeted Therapy

**Figure 3:**
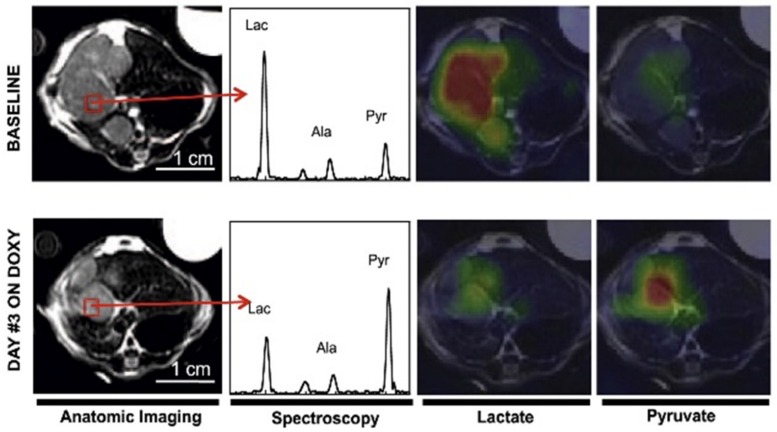
A representative example showing the application of hyperpolarized MRI and MRS in assessing response in liver tumor before and 72 h after MYC inhibition by Doxycycline (DOXY). A decrease in lactate is observed in the indicated voxel shown in red. Adapted from Reference [23] with permission.

Several targeted therapies were evaluated by measuring the conversion of hyperpolarized [1-^13^C]pyruvate to lactate [8,9,20,21,22,23]. LDH activity is highly dependent on the concentration and ratio of the reduced and oxidized form of the co-enzyme, Nicotinamide adenine dinucleotide (NADH/NAD^+^) [24,25]. A representative example showing the application of hyperpolarized MRI and MRS in assessing response to therapy is shown in Figure 3. During the reduction of pyruvate to lactate, NADH is oxidized to NAD^+^. Any targeted therapy that reduces this ratio will reduce pyruvate to lactate conversion. This has been observed in prostate cancer cell lines treated with protein kinase B (AKT) [26] and a NAMPT [27] (an enzyme necessary for NAD synthesis) inhibitor. The clinical use of HP-MR to assess treatment response compared the maximum pyruvate-to-lactate flux metric and apparent rate constant, k_PL_, in a prostate adenocarcinoma before and six weeks after the administration of androgen deprivation therapy [28]. The post-treatment flux value was 3.5-fold less than the pre-treatment value though there was minimal reduction in tumor volume. A six-month follow-up exam revealed significant clinical response with undetectable prostate-specific antigen (PSA) levels in the patient’s serum.

We have recently reported in vivo differences in pyruvate to lactate conversion between specific human prostate cancer cell lines [29] and prostate patient-derived xenografts (PDX) [30]. Higher conversion of pyruvate to lactate was observed in the prostate cancer cell line PC3 versus its metastatic subline PC3M. This correlated with higher lactate concentrations in the PC3 tumor tissue by ex vivo metabolic profiling. The PC3M cell line was found to be more dependent on glutaminolysis than its parent line and to be sensitive to glutaminase (GLS) inhibitor CB-839 while its parent line was not. This work was furthered by determining if hyperpolarized ^13^C pyruvate conversion could discriminate prostate PDX animal models into glycolytic and non-glycolytic subtypes. Four PDX models of prostate cancer characterized as androgen receptor (AR) positive or negative were imaged. No significant difference in pyruvate to lactate conversion between PDX models individually was observed even though each model was biologically unique [31,32,33]. However, a significance was observed between the conversion values when the subtypes were sorted AR+ versus AR- [30]. These studies show the overall ability of hyperpolarized pyruvate to lactate conversion to subdivide prostate cancer into specific phenotypes, which could be used to guide targeted therapy choice. We also tested the effect the GLS inhibitor CB-839 had on pyruvate to lactate conversion in an acute myeloid leukemia (AML) animal model [34]. A reduction in pyruvate to lactate conversion was observed just four hours after oral gavage of the compound.

The inhibition of the phosphatidylinositol 3-kinase (PI3K/Akt/mTOR) pathway with Everolimus was shown to correlate with a drop in hyperpolarized lactate levels in breast cancer and glioblastoma cells and xenografts [21]. The reduced appearance of lactate compared with pyruvate was attributed to a drop in LDH expression as a result of reduced levels of the transcription factor, hypoxia-inducible factor (HIF) 1α, which regulates expression of the LDH gene. A similar decrease in lactate-to-pyruvate ratios was observed in GS-2 glioblastoma rat models following treatment with Everolimus after seven days [35]. HP-MR also predicts treatment response to mTOR inhibitor (rapamycin) in patient-derived ccRCC xenograft models [36].

Fermentative glycolysis was targeted in with dichloroacetate (DCA) in an A549 lung cancer mouse model [37]. By inhibiting pyruvate dehydrogenase kinase (PDK), DCA activates pyruvate dehydrogenase (PDH) which shuttles pyruvate away from lactate production and into the Krebs cycle. This effect was observed through significantly reduced lactate production following the injection of hyperpolarized [1-^13^C]pyruvate compared to untreated mice. The MCT1 inhibitor, α-cyano-4-hydroxycinnamate (CHC) was administered to mice implanted with the mouse squamous cell carcinoma cell line, SCCVII [38]. Hyperpolarized [1-^13^C]pyruvate was injected, and a significantly reduced lactate-to-pyruvate ratio was observed in the treated tumors compared to those that were untreated.

Pre-treatment scans with hyperpolarized pyruvate can be useful for predicting the efficacy of certain therapies. In mice implanted with the human prostate cancer cell lines DU145 and PC3, the DU145-implanted mice displayed significantly higher conversion of pyruvate to lactate [39] compared to PC3. After both cohorts of mice were treated with the LDH inhibitor FX-11, this conversion significantly decreased in the DU145-implanted mice, but not in PC3. This was correlated with a significant reduction in the tumor growth rate in the DU145-implanted mice, but not in PC3. Thus, pre-treatment scans predicted an increased dependence on LDH, while post-treatment scans verified the efficacy of targeting LDH. The effects of FX-11 were also tested in a patient-derived pancreatic cancer xenograft model where the drug responder versus non-responder were identified by HP-MR before actual tumor shrinkage [9]. Furthermore, in a lymphoma xenograft model, HP-MR using pyruvate evaluated the LDH-A and glutaminase (GLS) inhibition by small molecule drugs FX11 and BPTES [bis-2-(5-phenylacetamido-1,2,4-diathiazol-2-yl) ethyl sulfide] respectively [8]. In all these investigations, a lactate-to-pyruvate flux ratio was used as a means to measure treatment responses. An alternative to the lactate-pyruvate ratio, which is critically dependent on the timing of injection and subsequent data acquisition, is to measure the lactate and pyruvate signals over time and fit these to a kinetic model [19,21,40]. In addition, fast spectroscopic imaging techniques can provide spatially resolved dynamic data of hyperpolarized pyruvate metabolism and spatially variable uptake of pyruvate. Spatially resolved changes in uptake and the pyruvate-to-lactate flux were observed in transgenic prostate tumors which were directly correlated to tumor cellularity and necrosis [41]. These findings highlight the value of this technique as a method to confirm drug delivery and drug target modulation before, or in the absence of, apoptosis and a reduction in tumor size.

The measurement of hyperpolarized pyruvate to lactate conversion is sensitive to a combination of enzymatic and transporter changes and does not always decrease as a result of treatment. In a provocative study, MCF-7 breast cancer and PC3 prostate cell lines were treated with the MEK (mitogen-activated protein kinase enzymes) inhibitor U0126 [42]. LDH activity was significantly increased in both cell lines, but the monocarboxylate transporter, MCT1, decreased in only the MCF-7 cell line and not in the PC3. As a result, hyperpolarized lactate production was significantly decreased in the MCF-7 breast cancer cell line but significantly increased in PC3 prostate cancer cell line following MEK inhibition. In these contexts, [^18^F]FDG-PET imaging can be potentially paired with HP-MR in such situations to provide excellent opportunities to interrogate therapeutic efficacy with glucose uptake in a multimodal metabolic imaging platform.

In addition to pyruvate, several other hyperpolarized substrates have been used to test the treatment efficacy of targeted therapies as shown in Table 1 and references therein. Hyperpolarized fumarate was used for detecting treatment response in tumors because the production of labeled malate would seem to be an unequivocal indicator of cell death [43,44,45]. An increased malate-to-fumarate ratio was reported in a breast cancer xenograft model after treating with the FDA approved drug, Sorafenib, which is a multi-kinase inhibitor [46]. Dehydroascorbic acid (DHA), a marker of cellular redox state [19,20], also showed promise for in vivo applications [47,48]. A number of new substrates are being tested as hyperpolarized imaging agents as listed in Table 1 [49,50,51].

Two other hyperpolarized Krebs cycle imaging probes have been developed, ^13^C labeled succinic acid [52] and its hydrophobic analog diethyl succinate [53,54,55]. These compounds have been polarized using both the DNP [56] and PHIP [52] polarization methods. These compounds convert to fumarate in vivo and in some cases to aspartate and malate. We observed changes in the conversion of hyperpolarized diethyl succinate 20 min after inhibitor 3-nitropropionate (a suicide inhibitor of succinate dehydrogenase) was administered [52]. This illustrates the ability of hyperpolarized succinate and its hydrophobic analog to measure the direct inhibition of enzymes involved in the Krebs cycle. In addition, we observed variable intratumoral metabolism and uptake with these two hyperpolarized compounds in mice bearing subcutaneous tumors of the kidney (RENCA), breast (4T1) or lymphoma (A20) [53].

### 3.2. Chemotherapy

Hyperpolarized [1-^13^C]pyruvate injections were used to evaluate the early response of rats orthotopically implanted with U-87 MG human glioblastoma cells following treatment with Temozolomide [57]. The ratio of lactate-to-pyruvate signals was significantly reduced at the one and two-day time-points when compared to untreated controls. The volume of treated tumors did not shrink until five days post-treatment.

The conversion of hyperpolarized [5-^13^C]glutamine to glutamate was measured before and after the administration of the natural anticancer drugs Resveratrol and Sulforaphane to DU145 and PC3 prostate cancer cells [58]. There was a significant reduction of glutamate production following treatment, which corresponded to a 50% reduction of cell count in each cell line. Hyperpolarized glutamine imaging was also exploited in IDH1 mutated murine GBM models [59,60]. Glutamine is a key metabolite in multiple metabolic pathways, and there is considerable interest in developing glutamine as a hyperpolarized imaging agent.

Tumors may respond to therapy by both apoptotic and non-apoptotic mechanisms including necrosis. Early detection of necrosis is of great interest in observing a tumor’s response to therapy. Hyperpolarized fumarate, a Krebs cycle metabolite, has been used to image necrosis in a murine EL-4 lymphoma model, with the formation of malate [43,45]. Following treatment with a chemotherapeutic drug Etoposide, there was a significant increase (2-3 fold) in malate production that correlated with tumor cell necrosis. The rate and amount of hyperpolarized malate production were significantly higher in the treated cells and tumors compared those that were untreated.

### 3.3. Radiation Therapy

In a C6 glioma rat model, conversion of hyperpolarized pyruvate to lactate in the tumors significantly decreased as soon as 72 h following radiotherapy, compared to pre-therapy values [61]. Interestingly, tumors continued to grow during this time, but long-term, these animals lived over threefold longer than untreated animals and gained weight while possessing rejuvenated activity and appetite. The efficacy of ionizing radiation in squamous cell carcinoma (SCCVII) and colon cancer (HT-29) was evaluated using hyperpolarized ^13^C-pyruvate MRI [62]. Thus, HP-MR could potentially be a useful tool to distinguish between true progression and pseudo-progression in tumors. Combined radiation therapy with conventional chemotherapy response on thyroid cancer was assessed by hyperpolarized pyruvate to lactate conversion [63]. The feasibility of using ^13^C metabolic imaging with [1-^13^C]pyruvate to detect early radiation treatment response in a breast cancer xenograft model was demonstrated in vivo and in vitro. Significant decreases in hyperpolarized [1-^13^C] lactate relative to [1-^13^C]pyruvate were observed in MDA-MB-231 tumors 96 h following a single dose of ionizing radiation [64].

### 3.4. Ablation Therapy

In transgenic adenocarcinoma of the mouse prostate (TRAMP) models, the prostate cancer was treated with high-intensity focused ultrasound (HIFU) ablation and imaged at several time-points [65]. The hyperpolarized pyruvate to lactate conversion was significantly reduced from pre-treatment values by three to four hours in the fully- and partially-ablated zones. However, at the one and five-day time-points, conversion remained significantly decreased in the fully ablated zones, which corresponded to cell death but had recovered in the partially-ablated zones. Thus, HP-MRI can be useful for both short-term and long-term treatment assessment.

### 3.5. Immunotherapy

Early assessment of cancer immunotherapy is an emerging area in diagnostic imaging. Cancer immunotherapy is employed by blocking the multiple negative regulatory proteins of T-cell activation known as cytotoxic T-lymphocyte-associated antigen 4 (CTLA4), programmed death-ligand 1 (PD-L1), and programmed death 1 (PD1) [66]. Immunotherapy is exceptionally successful in treating melanoma cancer patients; however, not all patients respond to this treatment. Therefore, being able to assess the immunotherapy response early would be beneficial in clinical care. Our laboratory has recently reported significantly different metabolic signature between immunotherapy resistant and sensitive murine melanoma tumors using in vivo ^13^C-MRS of hyperpolarized pyruvate and ex vivo ^1^H Nuclear Magnetic Resonance (NMR) spectroscopy [67].

**Figure 4:**
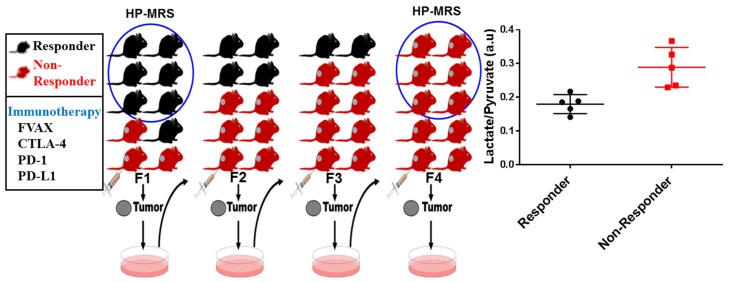
A schematic illustration of immunotherapy responding and non-responding tumors that are employed for hyperpolarized metabolic imaging trials and corresponding metabolic profiling.

B16 melanoma tumor-bearing C57BL/G RAG-1 knockout mice were treated with CTLA-4, PD-1, and PD-L1 blockade immunotherapy. The melanoma cancer cells extracted from non-responsive mice tumors in the first generation (F1) were injected into the next generation (F2) of mice and treated with immunotherapy, and this serial passage transfer continued up to the fourth generation (F4) as depicted in Figure 4. The initial mouse model in the first generation (F1) was described as immunotherapy sensitive, and the ones that were completely resistant to immunotherapy after the fourth serial passage of tumor cells were termed as immunotherapy resistant mouse models. These two cohorts of mice were picked up for HP-MR metabolic imaging trials. Pyruvate metabolism in melanoma mouse models treated with immunotherapy was well captured in real-time, and hyperpolarized pyruvate was readily metabolized to lactate. The quantitative ratio of lactate-to-pyruvate was determined and used as a treatment response marker of sensitive and resistant tumors. The lactate-to-pyruvate ratios were significantly higher in resistant tumors compared to sensitive tumors (*p* < 0.01). The differences in these ratios among the tumors were observed much sooner than the tumor volume shrinkage. In NMR metabolomics study on ex vivo tissue samples, a high level of lactate concentration in immunotherapy resistant tumors was observed which validates the in vivo results. This study demonstrates that metabolic imaging with hyperpolarized pyruvate and NMR metabolomics can distinguish immunotherapy resistant and sensitive tumors at an early time point. These exciting results also suggest that checkpoint blockade immunotherapy resistant tumors acquired hypermetabolic states with upregulated glycolysis to evade the immune response [67].

A table summarizing the different hyperpolarized compounds and their converted metabolites interrogating different enzymatic pathways are shown below.

## 4. Current Challenges for HP-MR Imaging Modality

For in vivo applications of metabolic imaging to be a reality, several important features are critical. Since hyperpolarized compounds agents are administered intravenously as a concentrated bolus (mM range), toxicity may limit the dose delivered. Transport of the agent into the cellular compartment or organ of interest is an important consideration along with the rate of metabolic conversion of the agent after it is internalized. In addition, the metabolic products of the hyperpolarized compound must have long longitudinal relaxation times (T_1_) to be observed in the time-scale of most HP-experiments (1–2 min). The current challenges in hyperpolarization research lie in improving methods for fast imaging [68], analyzing real-time kinetic data of metabolic conversion [69], interpreting the underlying biochemical meaning of these parameters, understanding the linkage of measured metabolite fluxes to underlying fluxes, establishing meaningful parameters from a radiological point of view, and translating new substrates to the clinic [70,71].

Despite all these challenges, GE Healthcare’s SPINLab polarizer has been approved for clinical use of DNP and has been used in Phase I trials for the diagnosis of prostate, brain, breast cancer, as well as in cardiac applications [72]. The first clinical trial with hyperpolarized ^13^C pyruvate was performed in patients with prostate cancer [73]. The trial revealed that ^13^C pyruvate was well tolerated by patients at high doses and was able to detect biopsy-proven tumor lesions. To date, over seven sites have performed clinical hyperpolarization trials using the SPINlab polarizer, and more than 20 such polarizers have been installed around the world.

## 5. Conclusion and Future Directions

Improved imaging of metabolism with hyperpolarization may enable precise clinical monitoring of metabolism in patients on specific therapies. As part of the comprehensive characterization of patients, this will lead to an improved prognostication, prediction, efficient development, and deployment of targeted metabolic therapies. The first clinical trial established the feasibility of imaging human tumors with hyperpolarized pyruvate. The power of metabolic imaging with hyperpolarized substrates is that it gives information on dynamic processes in real time. However, its weakness is that there is no data on the concentrations of the labeled substrates and, therefore, no quantitative information on flux expressed as molar concentration. Although measurements of changes in this apparent first-order rate constant have been demonstrated to be sufficient to detect treatment response in laboratory-based studies, this will need to be scrutinized in clinical studies. In summary, hyperpolarized MR based metabolic imaging technique has the potential to transform patient care by precisely and non –invasively assessing candidate “metabolic targets” before, during, and following disruptive metabolic interventions.

## Figures and Tables

**Table 1:** Hyperpolarized compounds and converted metabolites interrogating different enzymatic pathways.

Hyperpolarized Compounds	Downstream Metabolites	Active Enzymes	Selective Drugs	References
Pyruvate	Lactate, Alanine	LDH, ALT	FX11, Etoposide, Temezolomide, α-cyano-4-hydroxycinnamate, Everolimus, Rapamycin, DIDS, Immunotherapy (anti CTLA-4, PD-1)	[8,9,19,22,35,36,38,57,67]
Fumarate	Malate	Fumarase	Sorafenib, Etoposide	[43,44,45,46]
Glutamine	Glutamate	GLS	BPTES, CB-839, Resveratrol, Sulforaphane	[8,34,58]
Diethyl succinate	Fumarate	SDH	3-nitropropionate	[53]
Ketoisocaproate	Leucine	BCAT		[50,51]
Arginine	Urea	Arginase		[49]
Fructose	β-fructofuranose-6-phosphate, β-fructofuranose	Hexokinase, GLUT5		[10]
α-Ketoglutarate	2-hydroxyglutarate	IDH		[59,60]
Acetate	Acetyl-CoA, Acetyl- carnitine	Acetyl-CoA, Carnitine		[13,15]

## Abbreviations

HP-MR	hyperpolarized magnetic resonance
MRI	Magnetic resonance imaging
MR	Magnetic resonance
MRS	Magnetic resonance spectroscopy
NMR	Nuclear magnetic resonance
DNP	Dynamic nuclear polarization
PHIP	Parahydrogen induced polarization
SABRE	Signal amplification by reversible exchange
TRAMP	Transgenic mouse prostate cancer model
PDX	Patient derived xenograft
AML	Acute myeloid leukemia
LDH	Lactate dehydrogenase
NADH	Nicotinamide adenine dinucleotide
GLUT5	Glucose transporter type-5
ALT	Alanine transaminase
GLS	Glutaminase
IDH	Isocitrate dehydrogenases
BCAT	Branched-chain aminotransferases
PET	Positron emission tomography
FDG	Fluoro-deoxy-glucose
BPTES	Bis-2-(5-phenylacetamido-1,2,4-diathiazol-2-yl) ethyl sulfide
DIDS	4,4’-diisothiocyanostilbene-2,2’-disulfonic acid
HIF	Hypoxia-inducible factor
PSA	Prostate-specific antigen
HIFU	High-intensity focused ultrasound
k_PL_	Apparent rate constant
lac/pyr	Lactate-to-Pyruvate ratio
CTLA4	Cytotoxic T-lymphocyte-associated antigen 4
PD-L1	Programmed death-ligand 1
PD1	Programmed death 1
